# Phytol-based novel adjuvants in vaccine formulation: 1. assessment of safety and efficacy during stimulation of humoral and cell-mediated immune responses

**DOI:** 10.1186/1476-8518-4-6

**Published:** 2006-10-30

**Authors:** So-Yon Lim, Matt Meyer, Richard A Kjonaas, Swapan K Ghosh

**Affiliations:** 1 Department of Life Sciences, Indiana State University, Terre Haute, IN 47809, USA; 2 Division of Viral Pathogenesis, Department of Medicine, Beth Israel Deaconess Medical Center, Harvard Medical School, 330 Brookline Avenue, Boston, MA 02115, USA; 3 Indiana School of Medicine, Terre Haute, IN 47809, USA; 4 Department of Chemistry, Indiana State University, Terre Haute, IN 47809, USA

## Abstract

**Background:**

Vaccine efficacy depends significantly on the use of appropriate adjuvant(s) in the formulation. Phytol, a dietary diterpene alcohol, is similar in structure to naturally occurring isoprenoid adjuvants; but little is known of its adjuvanticity. In this report, we describe the relative safety and efficacy of phytol and its hydrogenated derivative PHIS-01 compared to commercial adjuvants.

**Methods:**

We tested adjuvant properties using a formulation consisting of either a hapten, phthalate-conjugated to a protein, keyhole limpet hemocyanin (KLH), or ovalbumin (OVA) emulsified with the test adjuvants in mice without any surfactant. Humoral immunity was assessed in terms of titer, specificity, and isotypic profiles. The effect on cell-mediated immunity was studied by assaying the induction of either OVA- or B-lymphoma-specific cytotoxic T-lymphocyte (CTL) activity.

**Results and Discussion:**

The phytol compounds, particularly PHIS-01, elicit increased titers of all major IgG subclasses, especially IgG2a. Unlike commercial adjuvants, both phytol compounds are capable of inducing specific cytotoxic effector T cell responses specific to both OVA and B-lymphoma tested. Phytols as adjuvants are also distinctive in that they provoke no adverse anti-DNA autoimmune response. Intraperitoneally administered phytol is comparable to complete Freund's adjuvant in toxicity in doses over 40 ug/mouse, but PHIS-01 has no such toxicity.

**Conclusion:**

These results and our ongoing studies on antibacterial immunity show that phytol and PHIS-01 are novel and effective adjuvants with little toxicity.

## Background

Designing effective vaccines depends not only on the nature of the antigens (Ag), but also on the inclusion of appropriate adjuvants to ensure optimum induction of protective immunity. The immunogenicity of a protein is inherently linked to its physico-chemical properties, but adjuvants can significantly influence the amplitude of the response. Traditionally, vaccines have consisted of attenuated/killed microorganisms, or isolated components. In recent years, vaccine formulations have included specific and safer recombinant proteins, synthetic peptides, and even vectored DNA [1,2]. In general, these vaccines are not as effective as those based on whole organisms, but the efficacy is often enhanced when used in conjunction with non-specific immunoadjuvants [3-5].

Adjuvant activity has been demonstrated in numerous natural products through serendipity and by trial and error [6,7]. However, in selecting adjuvants, their immunological properties are as important as their benefit-to-toxicity ratio. Adjuvants are often foreign to the body and thus capable of producing adverse reactions. These adverse effects can be a direct consequence of toxic or non-metabolizable components in their formulation or can result from the inclusion of agents that overstimulate the immune or inflammatory systems [8]. For example, CFA, which is used widely in experimental studies, produces excellent humoral and cell-mediated immunity, but is unsuitable for human and veterinary purposes because of toxicity. Hence, there is a need for identification of adjuvants that are both safe and efficacious.

The search for potentially useful adjuvants has often led to the use of isoprenoid compounds extracted from plant sources [9-12]. Because some of these compounds can be toxic, we considered developing isoprenoid adjuvants from substances that are common in the human diet. Epidemiological studies suggest that green vegetables in diets improve resistance to infection, and thus enhance immunity [13-15]. They may also help prevent some cancers by augmenting immunological responses against emerging neoplasms in the early stages of carcinogenesis [16-18]. Chlorophylls in green vegetables constitute an important source of an isoprenoid component, phytol (3, 7, 11, 15-tetramethyl-2-hexadecen-1-ol, C_20_H_40_O), a branched aliphatic alcohol, also present as the fatty acid side chain in tocopherols. Because phytols are hydrophobic, they are capable of interacting with the cell membrane. A number of recent studies have described various cellular and biological effects of phytol (19–21). However, there is as yet no definitive report on the adjuvanticity of phytol or any synthetic derivatives such as hydrogenated phytol or phytanol, named PHIS-01 (Patent pending) which has been studied in our laboratory.

In this report, we compared the adjuvant potential of both phytol and PHIS-01 to that of some commonly used adjuvants (Complete and incomplete Freund's adjuvants, TiterMax, Ribi's adjuvant system, and Alhydrogel). Since phytol and PHIS-01 are structurally similar to the mineral oil constituents in IFA and CFA, we included pristane for comparison as the protype mineral oil in this study. Most of these common adjuvants are not equally capable of enhancing either humoral and/or cellular responses against an immunogen. Moreover, their inclusion in vaccine formulations can engender adverse side effects, including the induction of anti-DNA antibody responses, the hallmark of lupus-like autoimmune disorders [22,23]. We demonstrate here that phytol, and to a greater extent phytol-derived PHIS-01, exhibit excellent adjuvanticity at low nontoxic doses and enhance an anti-hapten humoral response that consists of major IgG subclasses, especially IgG2a. They are equally capable of provoking anti-tumor cytotoxic T cell response. Moreover, unlike conventional adjuvants, phytol-derived PHIS-01 shows little toxicity or nephritogenic pathology resulting from induction of a cross-reactive anti-DNA antibody response. In our ongoing study, we have also noted that the phytol and PHIS-01 are superior adjuvants in eliciting anti-bacterial immune responses [24].

## Methods

### Immunological Studies

We studied the effects of commercial and experimental adjuvants on different immune parameters such as antigen-specific humoral responses, antibody isotypes, cell-mediated anti-tumor immunity, and autoimmune reactivity in BALB/c, C57Bl/6, and autoimmune-prone NZB mice. Gender-matched, 8–12 weeks old BALB/c and C57Bl/6 mice were bred in the animal facility of Indiana State University. To determine autoimmune parameters, six-week-old NZB/W F1 and NZB female mice (Harlan Sprague Dawley, Indianapolis, IN) were used. All animal experiments were performed according to guidelines of laboratory animal care (NIH publication 85-23), using specific protocols approved by the Animal Care and Use Committee (ACUC) of Indiana State University.

The commercial adjuvants used in this study consisted of CFA, IFA, Titermax, and RAS (Sigma Chemical Co., St. Louis, IL); phytol (Pfaltz and Bauer Inc., Waterbury, CT); and Alhydrogel (Accurate Chemical and Scientific Corp., Westbury, NY). Pristane (Sigma, St. Louis, IL) was also used for comparative assessment of plasmacytomagenic potential. Our experimental adjuvant consisted of phytol and a phytol derivative, PHIS-01 (patent pending). The latter was obtained by chemical reduction of phytol into phytanol following a published procedure [25].

### Anti-tumor vaccine efficacy

A B-cell lymphoma 2C3 was used in this study. We have extensively used this tumor model in previous studies [26-28]. This tumor, which secretes anti-phthalate 2C3-Ig, was generated from fusion of phthalate-KLH-primed BALB/c splenocytes with a non-secreting myeloma, X63-Ag8.653. Two other anti-phthalate hybridomas, designated as 1H5 and 3B4, which show high specificity for phthalate and DNA, were also previously described [27].

We also studied another tumor model, Ia-negative EL4 thymoma (H-2^b^) and EL4 cells transfected with ovalbumin (OVA)-cDNA gene (E.G7-OVA) obtained from American Type Culture collection (ATCC, Rockville, MD). Using this tumor model, we assessed OVA-antigen-specific CTL in C57BL/6 mice.

### Adjuvants on humoral response

Adjuvant effect on antibody response was studied in BALB/c (five or more in a group) which were injected intraperitoneally (IP) with phthalate-KLH conjugates (in BALB/c) emulsified in experimental or conventional adjuvants in a total volume of 400 μL (100 μg of each antigen). Control groups of mice were immunized with PBS only. Subsequent immunizations also contained adjuvants and were given at 10-day intervals. The mice were bled through retro-orbital veins five days after each immunization.

For assessment of antigen-specific cytotoxic effector activity, we used ovalbumin (OVA in 5 or more C57Bl/6 mice) also emulsified with adjuvants as above. We also assessed the efficacy of adjuvants in generating tumor-specific cytolytic response against the 2C3 tumor model in BALB/c mice. The latter group was repeatedly immunized with killed 2C3 tumor cells before spleens were dissected out for isolation and assessment of cytotoxic effector cells.

### Enzyme-linked immunosorbent assays (ELISA)

Indirect ELISA was performed to assess and correlate different humoral responses [26]. Serum antibodies were tested for their specificities to phthalate on polyvinyl 96-well flat bottom plates (Falcon) coated with either phthalate (as a conjugate of BSA) or calf thymus DNA. After the plates were blocked with 1% BSA/PBS O.N. at 4°C, various dilutions of sera (10–10000) were added to each well, and the plates were incubated for 1 hr at 37°C. The wells were washed with phosphate-buffered saline-containing 0.05% triton X, and rabbit anti-mouse Ig-HRP (50 μL) (at 1:3000 dilution) was added. Plates were incubated for 1 hr and washed again. Bound rabbit anti-mouse Ig-HRP was detected by addition of o-phenylene diamine (OPD) and hydrogen peroxide. The reaction was stopped with 50 uL of 10% H_2_SO_4_, and the color intensity was read at 490 nm.

### Generation of cytolytic effector cells

C57BL/6 mice were given three injections with OVA emulsified in test adjuvants. Spleen cells were obtained from C57Bl/6 mice on day 7 after the 3^rd ^immunization and prepared for ^51^Cr-release cytotoxicity assay [28]. For lymphoma, BALB/c mice were injected with each adjuvant 5 days before administration of live 2C3 tumor (5 × 10^6 ^cells/mouse). Splenocytes were harvested on day 8 and stimulated with killed 2C3 cells before ^51^Cr-release cytotoxicity assay.

Splenocytes were seeded into 6-well tissue culture plates at 6 × 10^6 ^cells/well in 2 ml RPMI/10% FBS, and then stimulated in vitro with killed E.G7-Ova cells or 2C3 cells (1.2 × 10^6 ^cells/well) for 5 days in the presence of 10% CO_2 _at 37°C to generate cytotoxic effector cells.

### Cytotoxicity assay

As previously described, the target cells were labeled at 37°C with 150 μCi of sodium ^51^Cr for 1 hr, washed three times in PBS, and then resuspended in RPMI/10% FBS [28]. The labeled target cells were then dispensed at 5 × 10^3 ^cells/well into 96-well plates. Effector splenocytes were added at various E:T ratios with appropriate target cells seeded in 96-well plates. The total volume of the reaction was 200 μL/well. The plates were incubated at 37°C for 6 h, after which they were centrifuged, and 30 μL of supernatant removed from each well was added to 96-well lumina plates to assess ^51^Cr release in a Top Count-NXT plate reader (Packard Instruments, Meriden, CT, USA). The percent specific lysis was determined by the formula: percent specific lysis = (sample release - spontaneous release/maximum release - spontaneous release) × 100. Spontaneous release never exceeded 18% of the maximum release. All cytolytic analyses described in this study were performed in triplicate and repeated at least three times in separate experiments. Specifically, the measurement of OVA-specific cytotoxic effector cell activity was performed using E.G7-OVA and EL4 cells as targets in C57Bl/6 mice, the latter serving as the negative control against OVA-specific effectors. For 2C3-lymphoma-specific cytotoxicity studies, 2C3 and a mastocytoma P815 were used as targets. P815 cells served as the negative control.

### Statistical analysis

The paired Student's t-test (Sigma Plot software) was used to determine statistical significance. Levels of p < 0.05 were considered statistically significant. Data are expressed as mean ± S.E.M.

## Results

### Generation of anti-phthalate antibody response in BALB/c mice

**Figure 1:**
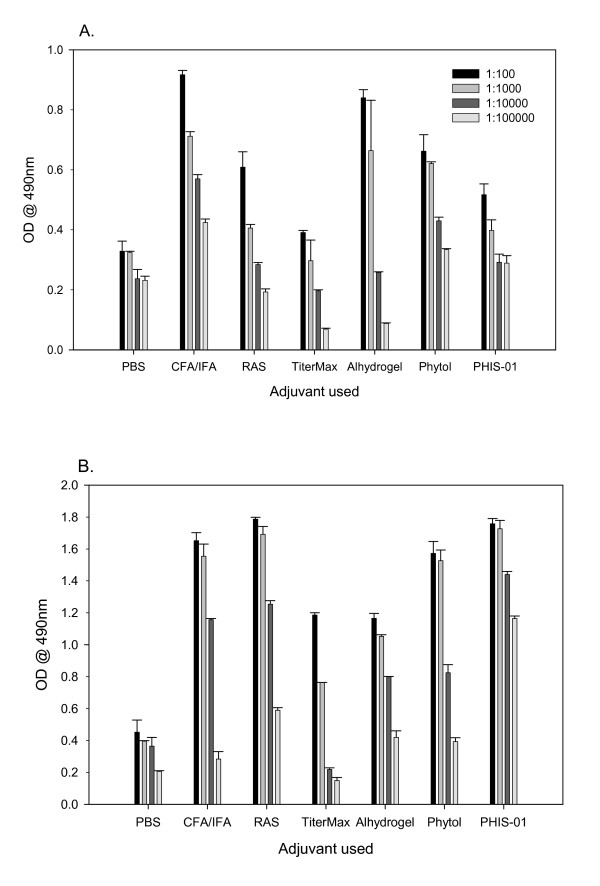
Anti-phthalate antibody response in BALB/c mice following vaccination with ortho-phthalate-KLH conjugate emulsified in various adjuvants. Serum samples were collected on day 5 after 1° (Fig. 1A) and 2° (Fig. 1B) immunizations and assessed by ELISA, as described. The results represent mean ± SD (n = 5 mice per group in two separate experiments).

Groups of 5 mice were injected with 100 μg of phthalate-KLH admixed with an adjuvant as described previously [27-29]. The commercially available adjuvants CFA, IFA, alhydrogel, pristane, TiterMax Gold, and Ribi Adjuvant System (RAS) were used in the preparation of immunogen according to the manufacturers' protocols. For phytol and PHIS-01, we adopted the protocol recommended for IFA/CFA. In order to compare adjuvanticity, mice were given identical doses of the antigen in each experiment. The efficacy of each adjuvant was evaluated by measuring serum antibody levels 5 days after each immunization. The results show that to a varying degree, all adjuvants tested augmented both 1° and 2° antibody responses to the phthalate conjugate (Fig 1A and 1B). There was little change in the magnitude of antibody responses in all groups of mice immunized during follow-up over a period of 2 months (data not shown). Interestingly, the 2° anti-phthalate antibody response was boosted as effectively by PHIS-01 and phytol adjuvants as by CFA/IFA combination or RAS. In contrast, TiterMax and Alum were ineffective (Fig. 1B).

### Effects of PHIS-01 and other adjuvants on induction of IgG subclasses

**Figure 2:**
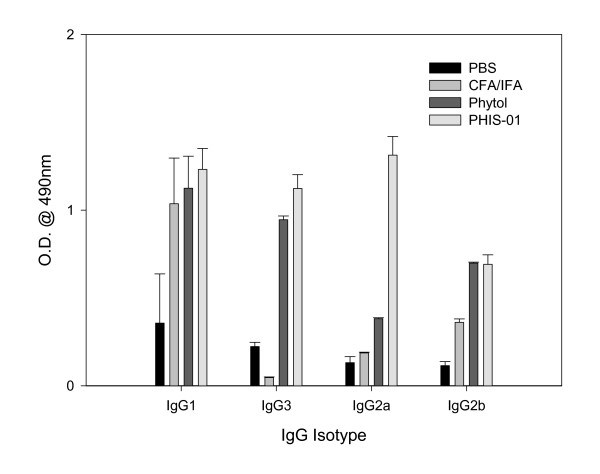
Assessment of classes and subclasses of phthalate-specific serum IgG antibodies induced in response to immunizations with ortho-phthalate-KLH conjugates in various adjuvants. The above serum samples were subjected to ELISA using commercial isotyping kits as described in Methods.

The effectiveness of a vaccine formulation depends to a large extent on the type of antibody subclasses induced, and adjuvants are known to play significant roles in vaccine efficacy. In this study, we determined by isotyping the effects of various adjuvants on induction of different IgG subclasses. Significant differences were indeed observed with the use of different adjuvants (Fig. 2). It is evident that all adjuvants tested favored IgG1 subclass; however, only PHIS-01 was also effective in induction of significant levels of IgG2a and IgG3 anti-phthalate antibodies suggesting a shift toward the Th1 type immune response. Evidently, the ratio of IgG1 to IgG2a Abs was <1 only in mice immunized with phthalate emulsified with PHIS-01.

### Induction of anti-tumor effector T cells

**Figure 3:**
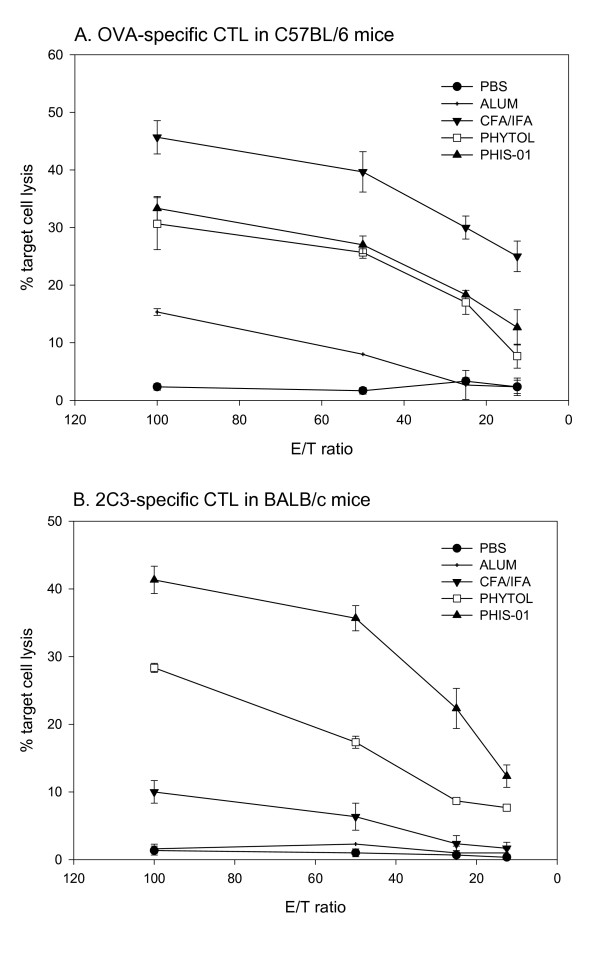
**Induction of tumor specific-cytotoxic effector responses**. Spleen cells were obtained from C57BL/6 mice on day 7 after 3^rd ^immunization with OVA in test adjuvants. BALB/c mice were given injection of test adjuvants 5 days before challenge with the B-cell lymphoma, 2C3 and sacrificed on day 8. Splenocytes harvested were stimulated with either killed E.G7-OVA or 2C3 cells *in vitro *for 5 days. Effector cells harvested on the fifth day were assayed in a ^51^Cr-release cytotoxicity assay as described under Methods. The results shown represent the mean of triplicates ± SD from two separate experiments (n = 3 mice per group/experiment). A. OVA-specific CTL response from C57BL/6 mice. B. Tumor-specific CTL response from BALB/c mice

Since induction of cytolytic effector cells is pivotal in ensuring the success of tumor vaccines, we investigated relative efficacy of phytols and known adjuvants in their ability to augment antigen-specific CTL activity in C57BL/6 and BALB/c mice using two different tumor models, E.G7-OVA and 2C3 respectively. Splenocytes from C57Bl/6 mice immunized with OVA emulsified in various adjuvants were stimulated in vitro with killed E.G7-OVA tumor cells and then evaluated for cytolytic activity against E.G7-OVA and untransfected EL4 cells. The results in Fig. 3A clearly show that phytols, unlike Alum, in vaccine formulation could elicit tumor-specific cell-mediated effector activity, albeit to a lesser degree than CFA/IFA. This effector-population was antigen-specific, as untransfected EL4 cells were not lysed (data not shown).

We previously reported induction of idiotype-specific cytotoxic T-lymphocytes (CTL) in BALB/c mice following prophylactic immunization with killed 2C3 tumors or during early stages of 2C3 tumor growth in vivo [30]. Using this model, we investigated whether this tumor-specific CTL response is augmented by injection of adjuvants and live 2C3 cells. As shown in Fig 3B, splenocytes of mice injected with phytol and particularly, PHIS-01 exhibited significant CTL response against 2C3 tumor target cells. These splenocytes had no cytotoxic activity against antigen-negative control tumors P815 (data not shown). In contrast, the commercial adjuvants CFA/IFA or Alum were ineffective against 2C3 B-lymphoma (Fig. 3B).

### Evaluation of toxicity and safety of phytol adjuvants

**Figure 4:**
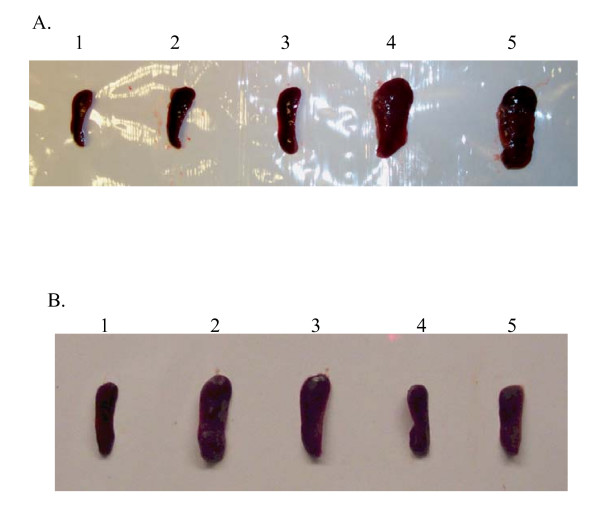
**Demonstration of splenomegaly in mice treated with different adjuvants**. Groups of 3–4 BALB/c mice were intraperitoneally injected with each adjuvant, and after 5 days their spleens were dissected out for observation. A representative result is shown below: (A) Effects ofvarious adjuvants on spleen size: 1. Spleens from mice injected with PBS. 2. Spleen from mice injected with Pristane. 3. Spleen from mice injected with IFA. 4. Spleen from mice injected with CFA. 5. Spleen from mice injected with Phytol. (B) Effects of different doses of phytols on spleens: 1. Spleen from mice injected with 100 μl of PBS. 2. Spleen from mice injected with 80 μg of Phytol. 3. Spleen from mice injected with 40 μg of Phytol. 4. Spleen from mice injected with 80 μg of PHIS-01. 5. Spleen from mice injected with 40 μg of PHIS-01

Adjuvants in general enhance interactions between innate and acquired immunity by mobilizing and activating the former, possibly by promoting danger signals [31,32]. In order to assess relative toxicity or inflammatory effects of the phytol and PHIS-01, we administered them in various concentrations (40–100 μg) via intraperitoneal routes to mice weighing about 20 g. Mice were weighed prior to treatment and at regular intervals thereafter throughout a period of one week, and then sacrificed to examine the major organs, such as liver and spleen. As shown in Table 1, the LD_50 _of PHIS-01 was much greater than 8 mg/kg bodyweight in mice, whereas all mice injected with the same dose of phytol were dead within 4 days. The difference between the body weight gain/loss in the test and control animals was less than 10% among groups of mice injected with <40 μg of phytol or PHIS-01. Furthermore, phytol induced splenomegaly comparable to that seen in mice treated with CFA (Fig 4A and 4B), but there was no sign of splenomegaly with 40–80 μg of PHIS-01 (Fig. 4B). Average spleen weights and cell numbers for each group of mice were reported in Table 2.

**Table 1:** Comparison of intraperitoneal lethal doses (LD_50_) and body weights of control mice and those injected with phytol and PHIS-01.

Test Adjuvants	Dose (μg)	Acute intoxication (% Survival in 24 h)	Mean body weight loss (%)				LD_50 _(mg/kg body weight)
				
			Day 1	Day 3	Day 5	Day 7	
PBS (Control)	250 (μl)	100	3.1	0	0	0	*ND
Phytol	40	100	13.86	19.41	24.75	15.92	>4
	80	100	14.1	8.7	7.8	All dead	
	100	50	13.98	26.73	All dead		
PHIS-01	40	100	3.8	10.16	9.08	8.9	>8
	80	100	7.1	17.69	13.5	10.06	
	100	100	7.8	13.68	22.92	16.92	

* Not detected

**Table 2:** Average weights and cell numbers of spleens from mice treated with different adjuvants.

Mouse Group	Spleen Weight (mg)	Cell Numbers/Spleen (× 10^7^)
Mouse injected with PBS	90.2 ± 5.6	6.5 ± 1.2
Mouse injected with Pristane	110.5 ± 6.4	7.6 ± 0.9
Mouse injected with IFA	160.3 ± 1.3	8.3 ± 0.5
Mouse injected with CFA	642.3 ± 2.4	35.6 ± 2.3
Mouse injected with Phytol (80 μg)	665.6 ± 5.7	42.4 ± 2.2
Mouse injected with PHIS-01 (80 μg)	141.3 ± 3.2	8.1 ± 1.2

All data are expressed as mean ± SD (n = 3 per group in two separate experiments).

### Induction of lupus-autoantibodies by adjuvants

**Figure 5:**
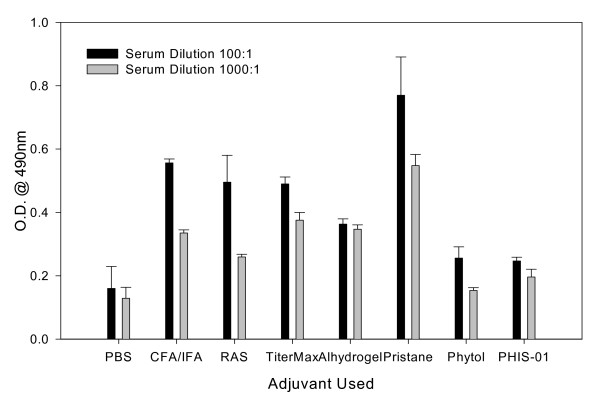
**Induction of autoreactive anti-DNA Ab responses**. Groups of BALB/c mice were immunized with phthalate-KLH emulsified in each adjuvant three times at 10 day-intervals. Their serum titers of anti-DNA antibodies were performed on ELISA plates coated with calf thymus DNA.

Although adjuvants such as CFA/IFA, or even pristane, effectively augment the immunogenic potentials of weak vaccines, they also induce lupus-type autoantibodies in most normal strains of mice [22,23]. It is not known whether this ability to induce lupus is unique to mineral oil adjuvants. To determine whether phytol products also induce lupus-type autoantibodies, we tested sera of phthalate-immunized BALB/c, NZB, and lupus-prone NZB/W F1 mice for cross-reactive anti-DNA responses. We previously reported such responses using adjuvants such as CFA and IFA [27-29]. As shown in Fig. 5, BALB/c mice immunized with phthalate in pristane induced high levels of anti-DNA autoantibodies after the 3^rd ^immunization. Similar autoantibodies were also produced, albeit at lower levels, by mice immunized with CFA/IFA and RAS. However, both phytol and PHIS-01 had no significant effects on the production of anti-DNA Abs.

### No signs of glomerulonephritis in phytol-treated mice

Further evaluation of safety and toxicity was assessed by histopathology of the kidneys from mice treated with phytol, PHIS-01 and IFA/CFA. Blood urea nitrogen (BUN) and proteinuria of autoimmune-prone NZB/W F1 mice immunized with phthalate-KLH in phytols or IFA/CFA were tested for by using Azostix (Bayer, Elkart, IN) and Multistix (Bayer, Elkart, IN) respectively. As described previously [27], mice immunized with phthalate in IFA/CFA reveal almost 3–4-fold higher BUN and urinary protein level indicating severe nephritis than those of control mice; however, no such kidney pathology was observed using phytol or PHIS-01 as adjuvants (data not shown).

### Ascites production in BALB/c mice using pristane and phytols

Mineral oils, pristane in particular, have been shown to promote ascites formation and induction of plasmacytoma in BALB/c mice [33-36]. To ascertain whether phytol and PHIS-01 exert similar effects, BALB/c mice were primed intraperitoneally with pristane, phytol, or PHIS-01. In contrast to pristane, phytol and PHIS-01 exhibited no plasmacytomagenic properties in preliminary studies. Nonetheless, as shown in Table 3, phytol was found to be comparable to pristane as a primer for propagation of hybridoma lines in vivo.

**Table 3:** Ascites production from syngeneic BALB/C mice using various priming agents

Hybrid line	Isotype of Ig	^1^Priming agent	Total volume collected (ml)	Mouse number providing ascites/number injected	^2^Yield/mouse (ml)	^3^Antibody titer (OD @ 490 nm)
2C3	IgG1 (γ1, κ)	None (PBS)	None	0/4	None	^4^ND
		Pristane	12	4/4	3	1.3
		Phytol	1.6	1/4	1.6	1.1
		PHIS-01	8	3/4	2.7	0.9

1H5^24^	IgM (μ, κ)	None (PBS)	None	0/4	None	^4^ND
		Pristane	16	4/4	4	0.8
		Phytol	9.3	2/4	4.65	0.75

2B4^24^	IgM (μ, κ)	None (PBS)	None	0/4	None	^4^ND
		Pristane	15	3/4	5	1.15
		Phytol	19	4/4	4.75	1.32

^1 ^40 μg of each substance was used

^2 ^Average volumes of ascites producing per number of mouse injected

^3 ^Ascites after salt fractionation using 50% ammonium sulfate were tested by ELISA at 50 μg/ml [31].

^4 ^Not detected; No significant antibody titer was detected.

## Discussion

The importance of safe and effective adjuvants in vaccine research cannot be overstated, and there is a growing need, not only for new vaccines but for new adjuvants as well. Most newly developed vaccines are based on selected target antigens consisting of single molecules or fragments derived from infectious microorganisms, or tumor cells. They are administered in the form of purified proteins, synthetic peptides, or vectored DNA. Such vaccines are usually poorly immunogenic and costly and/or difficult to produce. Moreover, many widely used vaccines can lose their effectiveness due to repeated use and for other biological reasons. Adjuvants can override such immunological inadequacy and help mount effective immune responses. Although in the past most vaccines have been designed to stimulate antibody responses, vaccines currently in development are increasingly designed to elicit cellular immune responses involving Th1 cells, and CTLs. Such responses are required to control chronic infectious diseases associated with viruses and intracellular pathogens, and also for the development of therapeutic vaccines against cancer.

In this study, we determined the adjuvanticities of chlorophyll-derived phytol and its chemically reduced derivative, PHIS-01, relative to those of commonly used commercial adjuvants. In the first study, mice were immunized with a hapten, phthalate, conjugated to KLH in one of the several adjuvants: phytol, PHIS-01, CFA, IFA, pristane, TiterMax, Ribi adjuvant system, and Alhydrogel or alum. Effectiveness was measured in terms of quantity, specificity, duration, and isotype of Abs generated. In another experiment, phytol, PHIS-01, CFA, and IFA were used to study induction of cell-mediated immunity, especially tumor specific CTL response to either OVA-transfected EL4 thymoma or 2C3 lymphoma in C57Bl/6 and BALB/c mice respectively. In addition, this study also addressed the issue of safety relative to efficacy of phytol-based adjuvants. Safety evaluation has been performed from the perspectives of toxicity, and the ability to induce adverse autoimmune reaction and plasmacytoma formation.

In this report, phthalate-protein conjugate was selected as the immunogen because of our previous finding that the anti-phthalate antibody response induced with IFA as the adjuvant elicits cross-reactive anti-DNA antibodies engendering lupus-like syndromes with kidney pathology [27-29]. We also reported that this adverse cross-reactivity is exacerbated by many commonly used adjuvants. Assessment of anti-phthalate and cross-reactive anti-DNA antibody responses in the presence of various adjuvants is thus a novel approach for evaluating the safety and effectiveness of adjuvants. In this investigation, we observed that phytol and PHIS-01 effectively enhance the immunogenicity of phthalate-conjugate without inducing anti-DNA antibodies. The mechanism underlying the suppression of this autoimmune reaction due to phthalates remains unclear.

Further evidence for the efficacy of phytol, and especially PHIS-01, as adjuvants, can be gleaned from the quality and levels of IgG responses elicited. PHIS-01-treated mice exhibited excellent anti-phthalate IgG2a response. This isotype is most desirable in therapeutic applications, because of its ability to activate complement cascades, and Ab-dependent cellular cytotoxicity which in turn ensures better protection against tumor or parasites. Moreover, induction of IgG2a is an indirect measure of the relative contributions of Th1 and Th2 cells. It remains to be determined whether this isotype switch reflects changes in cytokine milieu brought about by the phytol-based adjuvants.

In many instances, specific antigen-adjuvant combinations have been shown to promote antigen-specific production of Th1 type cytokines of (IFN-γ, IL-2) and cytotoxic T-cell responses [36-38]. However, there is as yet no specific combination that ensures sustained activity in terms of magnitude and duration of cell-mediated immune response. Our studies reveal that mice pre-treated with phytol, and especially PHIS-01, mount an effective CTL response recognizing lymphoma-associated Ig idiotype. Neither CFA/IFA nor alhydorgel appear to induce a similar response. However, when C57Bl/5 mice are immunized with soluble OVA and phytol or PHIS-01, cytotoxic effector activity of their spleen cells exhibit significant enhancement, although not as much as CFA/IFA.

In conclusion, phytol and PHIS-01 adjuvants appear to be more versatile as immunostimulants on the basis of their ability to promote effective humoral and cell-mediated immune responses. This is further evident in another study in which we assessed their adjuvanticity in engendering effective antibacterial responses [24]. In terms of toxicity, PHIS-01 induces little, if any, splenomegaly, implying no significant pro-inflammatory effects, and therefore is more useful than phytol. Further, only small amounts of phytol and PHIS-01 are required to stimulate immune responses. None of these two compounds stimulates reaginic immune responses, nor induces autoimmune lupus-like syndromes. Most importantly, phytol and PHIS-01 support hybridoma propagation in vivo without inducing formation of granulomatous tissue on peritoneal surfaces, which is a problem with pristane. Also, unlike pristane, these novel adjuvants have no effect on plasmacytoma development in BALB/c. In future studies, we plan to determine whether or not the differences in efficacy are due to a distinct cytokine milieu generated by these compounds

## Acknowledgements

The authors thank Professors William Brett and Jim Hughes of the Department of Life sciences and Tista Ghosh, MD, MPH, Tri-County Health Dept, Denver for their valuable suggestions and critical reading of this manuscript. This work was supported by grants from University Research (UNR215) and Indiana Academy of science (SAC131) (to S. G.) and Graduate Student funding from Indiana State University (to S-Y L.).
