# Correction: Phytol-based novel adjuvants in vaccine formulation: 1. assessment of safety and efficacy during stimulation of humoral and cell-mediated immune responses

**DOI:** 10.1186/1476-8518-5-3

**Published:** 2007-02-22

**Authors:** So-Yon Lim, Matt Meyer, Richard A Kjonaas, Swapan K Ghosh

**Affiliations:** 1 Department of Life Sciences, Indiana State University, Terre Haute, IN 47809, USA; 2 Division of Viral Pathogenesis, Department of Medicine, Beth Israel Deaconess Medical Center, Harvard Medical School, 330 Brookline Avenue, Boston, MA 02115, USA; 3 Indiana School of Medicine, Terre Haute, IN 47809, USA; 4 Department of Chemistry, Indiana State University, Terre Haute, IN 47809, USA

## Abstract

This is a correction article.

After the publication of the study [1] we noticed a substantial error in our published article. In the original article [1] the dosage units were erroneously described in μg instead of mg. All references to μg quantities should be replaced with mg quantities throughout the manuscript. Also the LD50 should be g/kg instead of mg/kg.

Thus, in the abstract section:

The 3^rd ^paragraph, 4th sentence, under the caption: Results and Discussion: one should read 40 mg/mouse instead of 40 μg/mouse.

In the results section:

The 5^th ^paragraph, 2^nd^, 5^th ^and 6^th ^sentences, under the caption: Evaluation of toxicity and safety of phytol adjuvants: replace μg with mg (in three places),

The 5^th ^paragraph, 4th sentence, under the caption: Evaluation of toxicity and safety of phytol adjuvants: replace 8 mg/kg with 8 g/kg

Figure 4 Legend, (under the caption: Demonstration of splenomegaly in mice treated with different adjuvants), lines 6 & 7: replace μg with mg (in four places)

Table 1 (next line beneath the table heading): Dose (mg, NOT μg), and LD50 (g/kg instead of mg/kg).

Table 2 (counting the Table heading): lines 6 and 7: (80 mg NOT 80 μg)

Table 3: Footnote (first): ^1^40 mg (NOT μg) of each substance was used.

Correction of the above errors in units does not alter the basic findings or conclusions of the original article.
